# Determination of Electroacupuncture Effects on circRNAs in Plasma Exosomes in Diabetic Mice: An RNA-Sequencing Approach

**DOI:** 10.1155/2019/7543049

**Published:** 2019-09-24

**Authors:** Yin Shou, Li Hu, Weibo Zhang, Yuan Gao, Ping Xu, Bimeng Zhang

**Affiliations:** ^1^Department of Acupuncture-Moxibustion, Shanghai General Hospital, Shanghai Jiao Tong University School of Medicine, Shanghai 200080, China; ^2^Acumox and Tuina Research Section, College of Acumox and Tuina, Shanghai University of Traditional Chinese Medicine, Shanghai 201203, China

## Abstract

circRNAs are involved in diabetes mellitus pathogenesis. Electroacupuncture (EA) is an effective therapeutic strategy for diabetes mellitus. However, whether the mechanism of action of EA on diabetes mellitus is related to altered circRNAs is unclear. The aim of this study was to reveal the effect of EA on circRNA expression in plasma exosomes and the underlying signaling pathway in mice with type 2 diabetes mellitus (T2DM). In total, 10 mice were randomly categorized into a normal group and 20 mice were used for the T2DM model preparation and randomly divided into the model and model + EA groups. Mice in the model + EA group were administered EA treatment. Changes in the fasting blood glucose (FBG) level and islet structure were evaluated. Plasma exosomes were subjected to RNA sequencing, and then bioinformatics analysis and real-time quantitative PCR (qPCR) verification were performed. EA treatment reduced the FBG level, preserved the islet structure, and reduced the islet *β* cell apoptotic rate in T2DM mice. After EA treatment, 165 differentially expressed circRNAs were found. GO and KEGG analyses revealed that thyroid hormone signaling was actively regulated by EA. circRNA/miRNA interaction analysis revealed mmu-mir-7092-3p to be closely associated with circINPP4B, suggesting that the phosphatidylinositol signaling pathway may be affected by EA. qPCR confirmed that 12 circRNAs had significant differences. These findings suggested that EA intervention can significantly protect islet function and improve the FBG level in T2DM, possibly via regulation of thyroid hormone and phosphatidylinositol signaling.

## 1. Introduction

Diabetes mellitus is a chronic endocrine disorder caused by lifestyle changes or inadequate insulin secretion due to hereditary factors, islet dysfunction, or development of insulin resistance. It often has some serious complications such as diabetic nephropathy, diabetic ophthalmopathy, and pathological changes in the cardiovascular system and can endanger human life and health [1]. The International Diabetes Federation estimated that there were 451 million diabetic patients worldwide in 2017 and if no measures are taken, the number will increase to 642 million by 2040 [2]. At present, treatment of T2DM includes self-management, weight loss, dietary adjustment, exercise, and medicine. Drug therapy is effective but has many side effects [3–6]. Because of its tolerable and unstable efficacy, it is difficult to determine their optimum doses for maintaining healthy blood sugar levels and weight [7–9].

Electroacupuncture (EA) is an integral part of traditional Chinese medicine and has been used to treat human illnesses for at least the past 3000 years. Acupuncture is reported to promote weight loss [10, 11]. In a statement issued by the American Diabetes Association (ADA) in 2005, acupuncture therapy was suggested to be effective for neuralgia at any stage of type 2 diabetes [12, 13]. In a single-blind experiment, acupuncture was found to significantly alleviate diabetic gastroparesis [14]. Acupuncture is also effective in treating diabetes mellitus with myasthenia gravis [15] and that with lower extremity arterial disease [16]. Recent experimental studies have shown that acupuncture can treat T2DM by regulating insulin resistance, improving islet beta cell function, and alleviating endothelial dysfunction [17, 18], which may involve AMPK and P13K/Akt mTORC1 signaling [19–21].

Exosomes, extracellular nanovesicles (30–150 nm) of endocytic origin, have been found to transport many biological molecules such as DNA fragments, circRNAs, and micro(mi)RNAs to promote intercellular communication and have also been shown to regulate many pathophysiological processes including immune response, inflammation, and infection [22]. Among these biological molecules transported by exosomes, the role of circRNAs, which are noncoding RNAs that function as miRNA “sponges” in various diseases, in diabetes mellitus has gradually attracted scientists' attention in recent years. A previous study showed that 529 circRNAs were aberrantly expressed in diabetic retinopathy and were involved in diabetic retina pathogenesis [23]. Another study revealed that circHIPK3 played a role in diabetic retinopathy by blocking miR-30a function, causing increased endothelial proliferation and vascular dysfunction [24]. Additionally, it was reported that 247 circRNAs were dysregulated in T2DM patients with depression [25]. Recently, it was pointed out that reduced expression of hsa_circ_0056891, hsa_circ_0063425, and hsa_circ_0071336 is an independent predictor of T2DM and increased the risk of T2DM [26]. Although there have been a few reports about relation between circRNAs and diabetes mellitus, whether the mechanism of action of acupuncture on diabetes mellitus is related to altered circRNAs remain unexplored.

In this study, for the first time, we investigated the effects of EA on circRNA expression in plasma exosomes and the signaling pathway of T2DM *in vivo*. Our results may provide new insights for elucidating the mechanism of EA in treating diabetes mellitus.

## 2. Materials and Methods

### 2.1. Experimental Animals

In total, 30 male C57BL/6 mice weighing 18 ± 2 g were obtained from the Animal Laboratory Center, First People's Hospital Affiliated to Shanghai Jiaotong University. All mice were maintained in the Animal Laboratory Center of First People's Hospital Affiliated to Shanghai Jiaotong University in a controlled environment of 25 ± 2°C, relative humidity 55–70%, and 12-h light/dark cycle. The mice were housed 5/cage, with free access to food and water. All experimental methods were approved by the Animal Ethics Committee of the First People's Hospital Affiliated to Shanghai Jiaotong University.

### 2.2. Animal Model

All male C57BL/6 mice were fed with basic forage (purchased from the Animal Breeding Center of Shanghai First People's Hospital) for one week for adaptation. Then, 10 mice were categorized according to their body weight into the normal (N) group (fed with basic forage). The remaining 20 mice were prepared as the T2DM model by feeding with high-forage diets consisting of casein 22.8%, dextrin 17%, DL-methionine 0.2%, minerals 4%, sodium bicarbonate 10.5%, vitamins 1%, heavy tartaric acid choline 0.2%, sucrose 17.5%, soybean oil 2.5%, hydrogenated coconut oil 33.35%, and potassium citrate 0.4%. After 6 weeks, these 20 mice were intraperitoneally injected with 1% streptozotocin (STZ, Sigma-Aldrich) solution (STZ dissolved in sodium citrate buffer) at a dose of 100 mg/kg daily for 7 consecutive days. For one week after the injection, fasting blood glucose (FBG) level and randomized blood glucose level was monitored. The model was considered unsuccessful if the randomized blood glucose level was less than 16.8 mmol/L or if the FBG level was less than 11.1 mmol/L. STZ was then injected at a dose of 150 mg/kg for the second time. The T2DM model mice were randomized into two groups: model group and model + EA group.

### 2.3. EA Intervention

After routine disinfection of acupoints, acupuncture Zusanli and Pishu points were stimulated on the mice in the model + EA group using needles with a diameter of 0.22 mm and length of 13 mm. After insertion of the needles, EA (frequency 2 Hz, intensity 1 mA–3 mA, Intermittent waveform, serial length 30 s, increasing once every 5 minutes, lasting for 15 minutes) was applied on the acupoints by connecting with a low-frequency pulse therapy instrument (G6805-2, Shanghai Medical Device High-tech Company, China), with slight shaking in the local muscle. The intervention was applied once every two days, 3 times a week, for 4 weeks, and 12 times in total.

### 2.4. FBG Measurement

FBG was measured before and after the modelling and after EA intervention for two weeks. After 16 hours of fasting, blood samples were collected from the tail vein of mice and applied to the test strip (lot: 4174080, Johnson & Johnson), with the test strip in the Johnson blood glucose meter. After 5 seconds, blood glucose values were read and recorded. To prevent infection, aureomycin eye ointment (purchased from Huaqing Pharmaceutical Company, Xinxiang, China) was applied to the tail wound on the day after testing.

### 2.5. Preparation of Pancreas Slices

At the end of the experiment, mice were anesthetized by intraperitoneal injection with pentobarbital sodium (60 mg/kg), and the whole blood of mice was extracted quickly with a 1 ml sterile syringe. The pancreas was quickly removed from the mice and washed with normal saline, dried with filter paper, fixed in 4% polyformaldehyde for 24 hours, embedded in paraffin, and sectioned for hematoxylin-eosin staining [27] and for the TDT-mediated dUTP-biotin nick end-labeling (TUNEL) to examine the apoptosis rate in islet beta cells [28]. The tissue and cell structure was observed under an optical microscope, and photographs were taken.

### 2.6. Extraction of Plasma Exosomes

Exosomes from blood plasma were isolated by differential centrifugation. Briefly, blood plasma (1 ml) was diluted with 6 ml PBS and differentially centrifuged at 1900 ×*g* for 10 min, 3000 ×*g* for 15 min, 500 ×*g* for 10 min, and 20000 ×*g* for 20 min at 4°C to eliminate cell debris, followed by centrifugation at 100000 ×*g* for 70 min. The resulting pellet was resuspended in 1 ml PBS, followed by centrifugation at 100000 ×*g* for 70 min at 4°C. The supernatant was removed, and the resulting exosomal pellet was reconstituted in 30 *μ*L of PBS and stored at −80°C.

### 2.7. Western Blotting

Exosomes were further characterized by western blotting. Protein concentration of exosomes was quantified using a BCA protein assay kit (Beyotime, China). The protein samples were separated on an 8% SDS-PAGE gel and transferred to polyvinyl difluoride (PVDF) membranes using a Mini Trans-Blot cell transfer apparatus (Bio-Rad, USA). After blocking with 5% BSA for 1 h, the membranes were washed 3 times (5 min each time) with 1x TBST and probed overnight with antibodies against CD63 (1 : 1000, Abcam), CD9 (1 : 2000, Abcam, USA), TSG101 (1 : 3000, Santa Cruz Biotechnology, USA), and calnexin (1 : 1000, Cell Signaling Technology, USA) at 4°C. The primary antibody was then discarded, and the membrane was washed 3 times (5 min each time) with 1x TBST and incubated with 1 : 2000 diluted secondary antibody at room temperature for 1 h and washed thrice with 1x TBST for 10 min each. The membrane was then covered with enhanced chemiluminescence (BOSTER) substrate and imaged in a gel imaging system (Bio-Rad).

### 2.8. Transmission Electron Microscopy

Transmission electron microscopy was used to visualize the morphological characteristics of exosomes. In total, 3 *μ*L of sample (1 mg/ml) was added onto a 300 mesh Formvar-coated copper grid and was fixed under an infrared lamp for 30 minutes. Samples not adhering to the copper grids were carefully removed with filter paper and then incubated in 2.5% glutaraldehyde phosphate buffer for 15 minutes. After rinsing with PBS and distilled water for 2 times, respectively, the samples were negatively stained with phosphotungstic acid and visualized using a Philips CM120 transmission electron microscope (Philips, Amsterdam, Holland) operated at 80 kV.

### 2.9. Nanoparticle Tracking Analysis

Exosomes were diluted and subjected to nanoparticle-tracking analysis (NTA) using a ZetaView PMX 110 (PMX, Germany) at 15000.00 US/cm conductivity, pH 7.4 electrolyte, and 27.34°C. The particle trajectory was recorded and the concentration and diameter distribution of the sample were output. The exosome concentration of the original solution was obtained according to the dilution.

### 2.10. Exosomal RNA Isolation

For RNA extraction, 20 *μ*L of exosome suspension was mixed with 1 ml Trizol buffer and placed on ice for 5 minutes. Then, 200 *μ*L of chloroform was added and the mixture placed on ice for 15 minutes after vigorous shaking for 30 seconds, followed by centrifugation at 13500 ×*g* for 10 minutes at 4°C. After the upper layer was transferred to a new enzyme-free tube, precooled isopropanol was added to this layer and mixed, and the mixture was kept at room temperature for 10 minutes. After centrifugation of this mixture at 13500 ×*g* for 10 minutes at 4°C, the supernatant was discarded and washed twice with 75% ethanol. The ethanol was then removed and the pellets were air dried for 15 minutes. Finally, 24 *μ*L of RNase-free water was added to the extracted RNA and stored at −80°C. The quantity and quality of the RNA were determined using the NanoDrop ND-1000 (Thermo Fisher Scientific, Waltham, MA, USA) and Agilent Bioanalyzer 2100 (Agilent Technologies, Santa Clara, CA, USA).

### 2.11. RNA Library Preparation and Sequencing

The rRNAs in total RNA were removed using Ribo-Zero rRNA Removal Kits (Illumina, USA). Then, TruSeqStranded Total RNA Library Prep Kit (Illumina, USA) was used to preprocess the RNA and construct the sequencing library. The quantity and quality of the RNA were determined using Agilent Bioanalyzer 2100 (Agilent Technologies, Santa Clara, CA, USA). According to the Illumina sequencing instructions, the 10 pM. library was denatured as a single-stranded DNA molecule, captured in an Illumina flowcell, amplified into clusters *in situ*, and sequenced over 150 cycles on an Illumina HiSeq sequencer using a two-terminal mode. The library was constructed and sequenced by Shanghai Yunxu Biotechnology Co., Ltd.

### 2.12. Annotation of Host Linear Transcripts and Identification of Differentially Expressed circRNAs

After sequencing on the Illumina HiSeq 4000 sequencer, paired-end reads were retrieved. Cut adapt [29] (v1.9.3) software was used to eliminate the low-quality reads and obtain high-quality reads. STAR software (v2.5.1b) [30] was used to compare the high-quality reads to the reference genome/transcriptome, and DCC software (v0.4.4) [31] was used to detect and identify circRNA. The identified circRNA was annotated based on the circBase database [32]. circRNAs with fold changes ≥2.0 or fold change ≤0.5 in expression level in the model + EA group compared with both the normal and model group identified as differentially expressed. Linear transcripts were annotated according to the location of the chromosome where the circRNA sequence was overlapped.

### 2.13. GO and KEGG Pathway Analysis of Linear Transcripts

To analyze the potential functions of linear transcripts, the identified linear transcripts sequences were mapped with Gene Ontology Terms (http://geneontology.org/). GO term matching was performed with blast2go and go2protein. Gene functions were classified into three subgroups, namely, biological process, cellular components, and molecular function. To analyze the statistical significance of GO terms, we employed hypergeometric tests. The top 10 enriched GO terms affected by EA ranked by enrichment score were presented.

To annotate the identified differential linear transcripts in each pathway, Kyoto Encyclopedia of Genes and Genomes (KEGG) pathway (http://www.genome.jp/kegg/pathway.html) analysis was conducted. To analyze the statistical significance of KEGG pathway enrichment, we employed hypergeometric tests. The top 10 pathway enrichment of upregulated and downregulated differentially expressed host linear transcripts affected by EA intervention were presented.

### 2.14. miRNA Target Prediction

In order to explore the function of circRNAs, putative interactions between differentially expressed circRNAs and their target miRNAs were theoretically evaluated using TargetScan and miRanda database. A hit between any expressed miRNA (including the new predicted miRNA) and a target circRNA was considered for a miRanda score of 140 or higher.

### 2.15. circRNA-miRNA Coexpression Network Analysis

Evidences have shown that circRNAs could bind with miRNAs and function as natural miRNA sponges to influence related miRNAs' activities. circRNA-miRNA coexpression network was built based on the prediction of miRNA-binding sites and the correlations between circRNA and miRNA. Two downregulated and three upregulated circRNAs were selected to generate a network map with cytoscape software (V. 3.2.1). Yellow nodes represented circRNAs and green nodes represented miRNAs.

### 2.16. Hierarchical Clustering Analysis

To generate an overview of differentially expressed circRNA profiles among the three groups, hierarchical clustering analysis was conducted based on the expression values of all target circRNAs and differentially expressed circRNAs using the Cluster and TreeView programs.

### 2.17. Quantitative Real-Time PCR

To validate the RNA sequencing data, we performed qPCR analysis of 12 differentially expressed genes. cDNA reverse transcription was performed from total RNA using the iScript Reverse Transcriptase Kit (Biorad, Hercules, CA). GAPDH was selected as a reference gene for all experiments. All primers for qPCR were synthesized by Sangon Biotech, China. The primer sequences used for qPCR are detailed in Supplementary [Supplementary-material supplementary-material-1]. qPCR was performed using the SYBR Green Supermix kit (Bio-Rad) per the manufacturer's instructions. After activation of the polymerase enzyme at 95°C for 10 min, 40 cycles of 95°C for 10 s, 60°C for 60 s, and 95°C for 15 s were performed on the Gene Amp PCR System 9700 (Applied Biosystems, Foster City, CA). Melting curve analysis was used to confirm the specificity of the amplification reactions. Relative gene expression was quantified in triplicate (*n* = 3). The relative expression levels were calculated using the 2^−ΔΔCT^ method.

### 2.18. Statistical Analysis

All data were processed using SPSS 19.0 software. Normal distribution data are expressed as mean ± SD and were analyzed using ANOVA. The skewed distribution data are expressed as median (*M*) and quartile spacing (*Q*1, *Q*2), and the rank sum test was used for intergroup comparison. *P* < 0.05 was considered significant.

## 3. Results and Discussion

### 3.1. General Information on Mice

There were 8 mice with random blood sugar level higher than 16.8 mmol/L and 1 with fasting blood sugar level higher than 11.1 mmol/L; and the success rate of the model was 45%. The remaining 11 mice were injected with STZ for a second time, with a 100% success rate. After high-fat feeding, the body shape of the mice changed, revealing central obesity and sparse hair. After STZ injection, the mice became depressed, showed decreased activities, had dry and sparse fur, excreted decreased urine volume, and consumed higher volumes of drinking water; all changes were significant.

### 3.2. Effect of EA Intervention on FBG Level

After modelling, the FBG levels in mice of the M group and model + EA group were markedly increased. After 4 weeks of EA intervention, the FBG levels of the M group and model + EA group were still remarkably higher than that of the N group (*P* ≤ 0.001) (Table 1). However, a significant reduction in FBG level was observed in the model + EA group when compared with that in the M group (Table 1). EA was thus suggested to significantly improve the FBG level although it could not restore the normal FBG level.

### 3.3. Effect of EA on Pancreatic Tissue and Islet *β* Cell Apoptosis Rate

**Figure 1:**
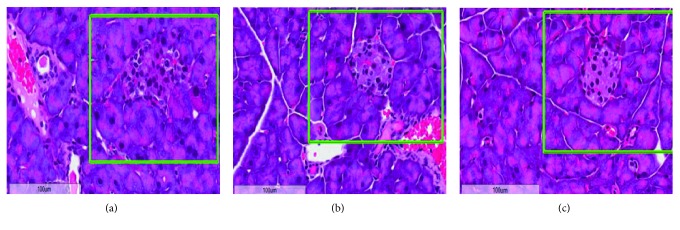
Hematoxylin-eosin staining in the pancreatic tissue of the model group (a), model + EA group (b), and normal group (c). Magnification: 400x. Islet structure is marked with a green box.

The pancreas is an important organ for secreting insulin and regulating the blood sugar levels. In order to study the effect of EA on pancreatic tissue in T2DM, hematoxylin-eosin staining was performed. As shown in Figure 1(a), the pancreas of normal mice showed intact islet structure, regular arrangement of pancreatic cells, and clear nuclei. In the M group, a large number of inflammatory cells infiltrated, surface adipocytes degenerated, interstitial hyperplasia, vasodilation, congestion, and necrosis were observed in the pancreatic tissue, with no complete islet structure (Figure 1(b)). In the model + EA group, mild and moderate inflammatory cell infiltration, vasodilation, and hyperemia were observed in the pancreatic tissue of mice and the structure of islets was preserved in the sections (Figure 1(c)). These results indicated that EA could effectively protect the pancreatic tissue in T2DM and inhibit the effect of STZ.

Further TUNEL analysis showed that the apoptosis rate of islet *β* cells in the model and model + EA groups was obviously higher than that in the N group (Table 2). Nevertheless, the apoptosis rate of islet *β* cells in the M + EA group was still significantly lower than that in the M group, implying that EA could significantly improve the apoptotic rate of islet *β* cells.

### 3.4. Identification of Differentially Expressed circRNA Profiles in Plasma Exosomes

**Figure 2:**
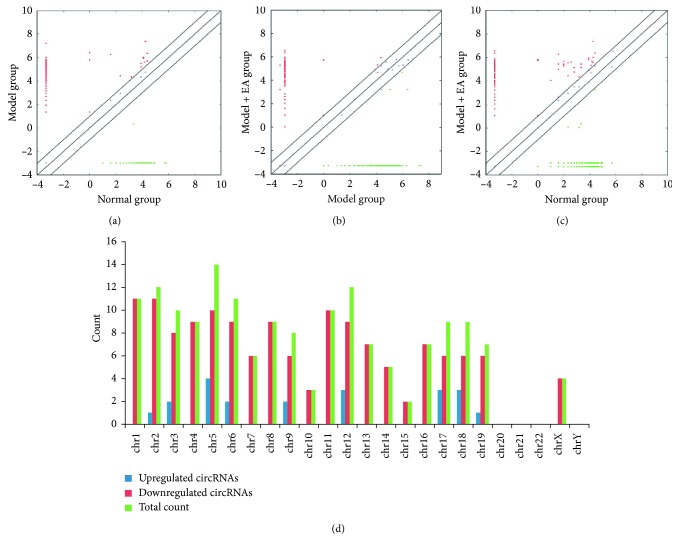
Differences and characterizations of circRNA expression profiles in plasma exosomes among the three groups. (a) Scatter plots between model and normal groups. (b) Scatter plots between model and model + EA groups. (c) Scatter plots between model + EA and normal groups. The values plotted on *X* and *Y* axes of scatter plots are the averaged normalized signal values of each group (log 2 scaled). The middle line refers to no difference between the two groups. The circRNAs above the middle line and below the middle line indicate more than 2.0-fold changes between two groups. (d) The distribution of differentially expressed circRNAs in the model + EA group in mouse chromosomes.

**Figure 3:**
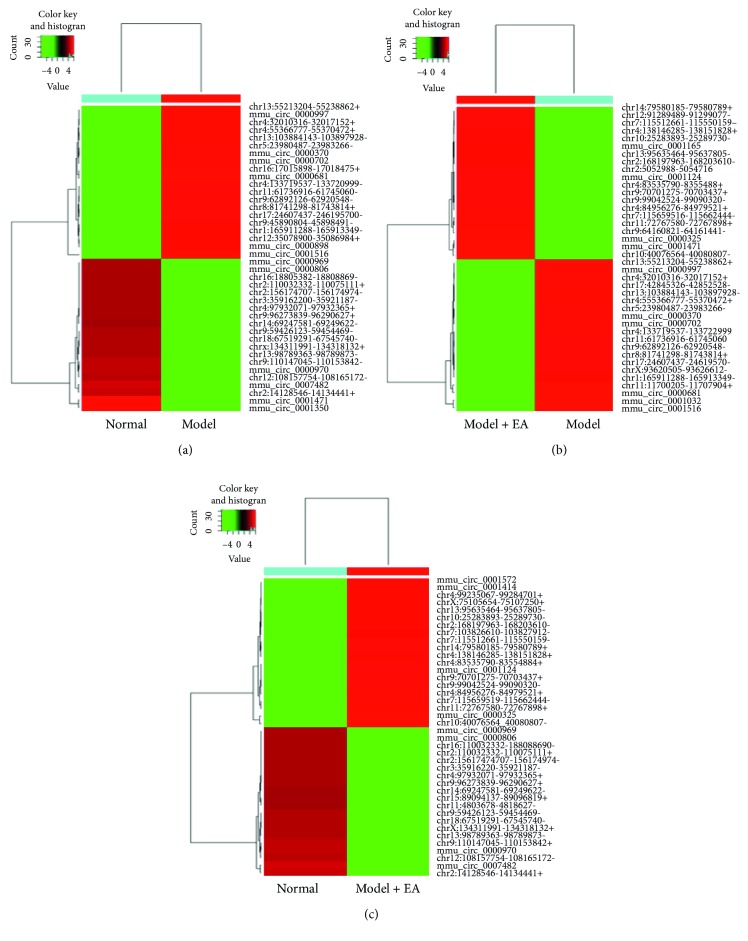
Heat map and hierarchical clustering showing expression values of all differentially expressed circRNAs among the three groups. Each column represents a sample and each row represents a circRNA. Red strip represents high relative expression and green strip represents low relative expression. (a) Hierarchical cluster analysis between model and normal groups. (b) Hierarchical cluster analysis between model and model + EA groups. (c) Hierarchical cluster analysis between normal and model + EA groups.

Plasma exosomes confirmed by western blotting, transmission electron microscopy, and NTA (Supplementary [Supplementary-material supplementary-material-1]) were subjected to RNA sequencing. As a result, a total of 579 circRNA targets were found in the plasma exosomes of three groups (Supplementary [Supplementary-material supplementary-material-1]). To provide a comprehensive landscape of the origination of circRNAs, linear transcripts of circRNAs from the corresponding genes were annotated and the circRNAs distribution in the genome was also explored according to the location of the chromosome where the circRNA sequence was overlapped (Supplementary [Supplementary-material supplementary-material-1]). Differentially expressed circRNAs were displayed through fold change filtering (Figure 2(a)–2(c), Supplementary [Supplementary-material supplementary-material-1]). Additionally, 165 circRNAs were detected to be differentially expressed in the M + EA group compared with the M and N groups. The distribution of the differentially expressed circRNAs in the M + EA group on the mouse chromosomes is shown in Figure 2(d). Among these, 21 circRNAs, which were downregulated in the M group when compared with the N group, were upregulated after EA treatment, whereas 144 circRNAs that were upregulated in the M group compared to the N group were downregulated after EA treatment. The top ten upregulated and downregulated circRNAs are listed in Table 3 by fold change. Hierarchical clustering analysis indicated that these differentially expressed circRNA expression pattern were distinguishable among three groups (Figure 3).

### 3.5. GO Annotation and Pathway Enrichment Analysis

**Figure 4:**
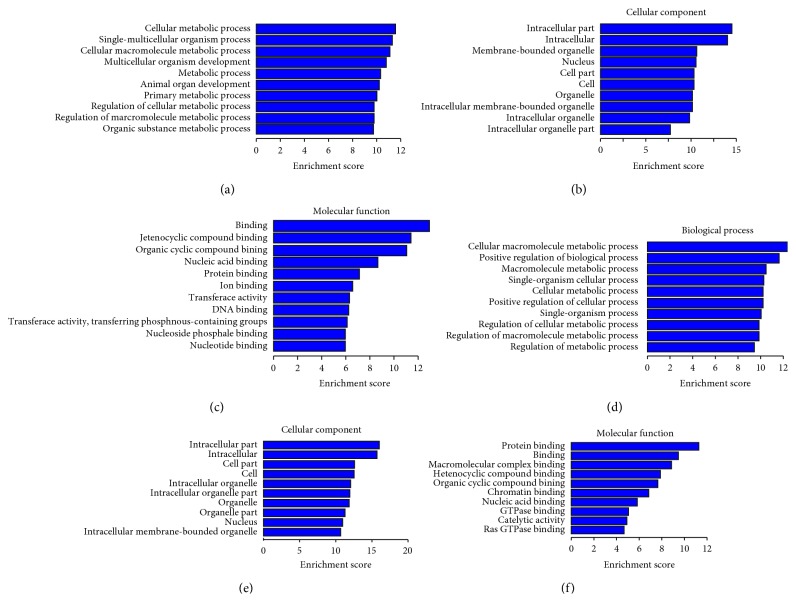
Gene ontology (GO) annotation of host linear transcripts affected by EA from the Gene Ontology Terms database (http://www.geneontology.org) compared to the normal (a, b, c) and model group (d, e, f). (a, d) Cellular components; (b, e) biological process; (c, f) molecular function.

Under the assumption that circRNA function would be related to the known function of the host linear transcripts, differentially regulated linear transcripts were further mapped with Gene Ontology Terms (http://geneontology.org/). According to GO annotations, all identified differentially expressed circRNAs among the three groups were divided into three categories: cellular components, biological processes, and molecular functions (Supplementary [Supplementary-material supplementary-material-1], Supplementary [Supplementary-material supplementary-material-1]). We employed a hypergeometric test, followed by FDR (false discovery rate) multiple correction to calculate the *P* value of each GO item for enrichment analysis. In total, GO items of differentially host linear transcripts affected by EA were involved in basic metabolism, cell metabolism, cell macromolecule metabolism, organic substance metabolism, cell growth, development, and other related functions (Figure 4). The top 10 GO annotations enriched for the upregulated and downregulated linear transcripts are displayed in Tables 4 and 5, respectively.

**Figure 5:**
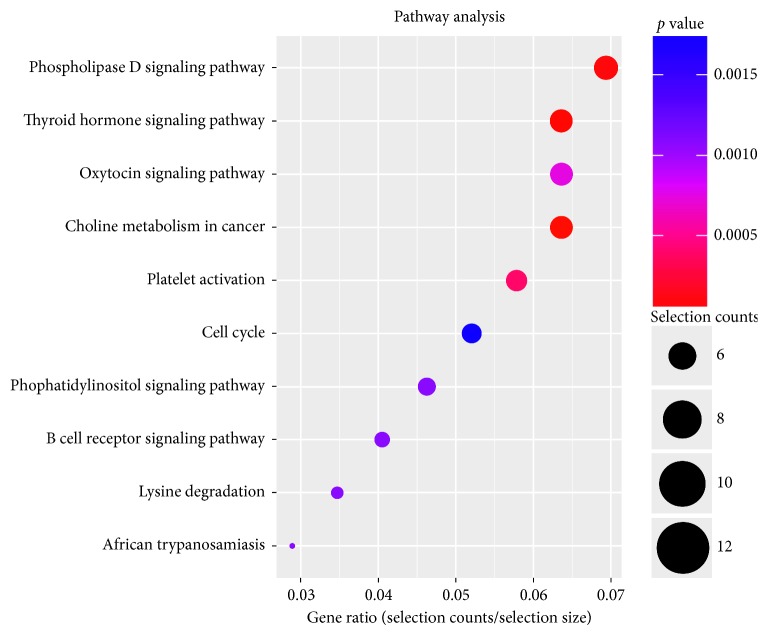
KEGG pathway enrichment of host linear transcripts affected by EA. The dot plot shows the gene ratio value of the top ten most significant enrichment pathways.

To obtain an overview of the main biochemical metabolic pathways, all differentially expressed host linear transcripts were analyzed using KEGG, to provide an alternative functional annotation of genes according to their related biochemical pathways (Supplementary [Supplementary-material supplementary-material-1]). Our analysis revealed that 165 differentially expressed host linear transcripts affected by EA treatment in T2DM were involved in a total of 56 annotated pathways (Figure 5). The upregulated host linear transcripts mainly belonged to the following pathways: phospholipase D signaling pathway, thyroid hormone signaling pathway, oxytocin signaling pathway, cancer choline metabolic pathway, platelet activation, cell cycle, phosphatidylinositol signaling system, B cell receptor signaling pathway, lysine degradation, and African trypanosomiasis. The top 10 results of upregulated pathway enrichment affected by EA treatment are listed in Table 6. On the contrary, the downregulated host linear transcripts mainly belonged to the following pathways: Fc*γ*R mediates the macrophage phagocytosis signal transduction pathway, HIF-1 signal transduction pathway, phosphatidylinositol signal system pathway, cancer choline metabolism pathway, lysine degradation pathway, NOD-like receptor signaling pathway, mTOR signaling pathway, phosphoinositol (IP) metabolism pathway, and cancer proteoglycan pathway. The top 10 results of pathway enrichment affected by EA treatment are listed in Table 7.

### 3.6. Prediction of circRNA/miRNA Interaction and Construction of an Interaction Network

**Figure 6:**
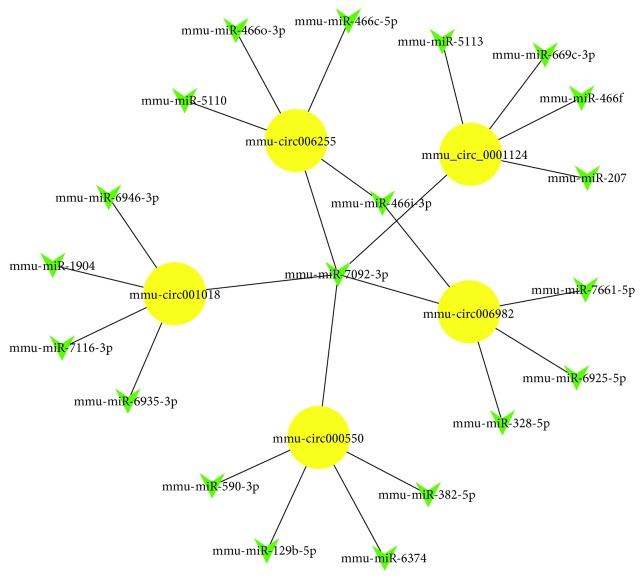
The circRNA/miRNA network analysis of two downregulated (yellow nodes) and three upregulated circRNAs (yellow nodes) with their acting miRNAs (green nodes).

circRNAs act as a “microRNA sponge” to fine-tune the levels of microRNAs. In order to explore the function of circRNAs, interactions between differentially expressed circRNAs and their target miRNAs were theoretically predicted by microRNA target gene prediction software according to the TargetScan and miRanda database. Supplementary [Supplementary-material supplementary-material-1] displays each circRNA and its potential complementary binding miRNAs. The predicted expression of top 10 upregulated circRNA-bound microRNAs and downregulated circRNA-bound microRNAs affected by EA are shown in Tables 8 and 9. Furthermore, an entire interaction network of two downregulated (mmu-circ000550 and mmu-circ001018) and three upregulated circRNAs (mmu_circ_0001124, mmu-circ006255, and mmu-circ006982) with their acting miRNAs were delineated using Cytoscape (Figure 6).

### 3.7. Validation of the Differentially Expressed circRNAs

**Figure 7:**
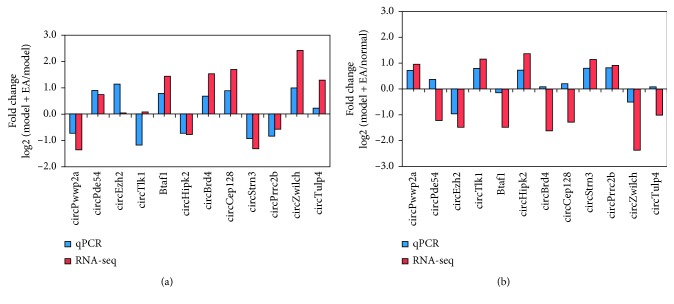
qRT-PCR validation for the expression of 12 circRNAs. Comparison between RNA-seq data and qPCR results. The vertical axis shows the fold change (log 2 transformed) of each circRNA between the model + EA and model groups (a) or the model + EA and normal groups measured (b) by qPCR and RNA-seq, respectively.

Twelve differentially expressed circRNA genes, namely, circPwwp2a, circPde5a, circEzh2, circTlk1, circBtaf1, circHipk2, circBrd4, circCep128, circStrn3, circPrrc2b, circZwilch, and circTulp4, were randomly selected and verified by qRT-PCR. The log 2 fold changes were calculated for RNA-seq data and qPCR results (Figure 7(a) and 7(b)). Most qRT-PCR results matched well with the RNA-seq data.

## 4. Discussion

It is well known that insulin resistance and beta cell dysfunction are the main pathogenic factors of T2DM. Previous studies have shown that EA can treat T2DM by regulating insulin resistance and improving the function of islet beta cells. The majority of acupoints selected by EA are Zusanli, Pishu, Weiwanxia Yu, and Zhongwan, among others [17, 18]. In this study, the Zusanli and Pishu points were selected for EA application, which resulted in an obvious decrease in FBG levels in the T2DM model mice. These results indicated that EA had a certain therapeutic effect on relieving hyperglycemia, which was consistent with the results of previous studies [17, 33, 34].

Some studies have been conducted with regard to the potential pathways by which EA exerts its therapeutic effect on diabetes mellitus. A previous study by Tominaga et al. [21] showed that repeated application of EA to the Zusanli is capable of improving diet-induced insulin resistance, probably through activation of AMPK signaling in skeletal muscles. Lan et al. [19] found that EA mitigates endothelial dysfunction via effects on the PI3K/Akt signaling pathway in high-fat-diet-induced insulin-resistant rats. On the contrary, Leng et al. [20] found that EA can improve obesity and reduce the potential risk of type 2 diabetes via hypothalamic Tsc1 promoter demethylation and by inhibiting the activity of mTORC1 signaling pathway. These results are in accordance with our results. We also found that EA treatment could upregulate the signaling pathways of thyroid hormone, sphingolipid biosynthesis (ganglion series), cGMP-PKG, cancer transcriptional error regulation, cancer choline metabolism, Rap1, actin cytoskeleton regulation, and adipocyte lipid regulation and could downregulate the Fc*γ*R-mediated macrophage phagocytosis signal transduction pathway, HIF-1 signal pathway, phosphatidylinositol signal system pathway, cancer choline metabolism pathway, lysine degradation pathway, NOD-like receptor signal pathway, mTOR signal pathway, phosphoinositol (IP) metabolism pathway, and cancer proteoglycan pathway. Our results suggest that the effect of EA on T2DM is multifactorial and is exerted at multiple levels. Regulating the blood sugar level may only be one of the smaller aspects of EA; other therapeutic effects of EA on T2DM still need to be explored.

Interestingly, we found that the effect of EA on the thyroid hormone signaling pathway is very prominent in T2DM, with both upregulated genes (MED13, MED13L, NCOA2, PIK3CB, and SLC16A10) and downregulated genes (MED12L, PLCG2, PRKCA, and TSC2) being observed. Previous studies have revealed the association of hyperthyroidism [35–37] and hypothyroidism [38–40] with insulin resistance in T2DM. Thyroid hormones could increase the concentration of free fatty acids, thereby enhancing the storage and oxidation of glucose and affecting insulin secretion [41, 42]. Previously, it was reported that 15 Hz EA at ST36 improves insulin sensitivity and reduces free fatty acid levels in rats with chronic dexamethasone-induced insulin resistance [43]. This suggested that EA may improve insulin resistance by regulating thyroid hormones in T2DM, which needs to be confirmed by further experiments.

In addition, we constructed an interaction network between circRNAs and microRNAs and found that mmu-mir-7092-3p, one of the most active miRNAs, was closely associated with circINPP4B, which was significantly downregulated by EA. INPP4B is involved in the phosphatidylinositol signaling pathway, which inhibits PI3K/Akt signaling and is emerging as a tumor suppressor in a variety of tissues [44]. The interaction network of circRNA/miRNA further confirmed that the effects of EA on T2DM may involve the PI3K/Akt signaling pathway.

This study has several limitations. First, only 1 replicate has been sequenced from each condition. Second, this study assessed differentially expressed circRNAs via fold changes ≥2.0 or fold change ≤0.5 in the expression level in the model + EA group compared with both the normal and model group. As only 1 replicate per condition was performed, the evaluation criteria of differentially expressed circRNAs cannot contain *P* value. Third, although the expression levels of selected circRNAs seem to change with EA induction, their specific functions have not been studied or verified. As for the circRNAs function as a sponge, further studies about the effects on the predicted miRNAs or the expression level of the proposed interacting mRNAs are still needed.

## 5. Conclusion

In conclusion, we found that EA intervention can regulate the thyroid hormone and phosphatidylinositol signaling pathway to attenuate apoptosis of islet *β* cells and protect islet function in T2DM mice. This study opens up new avenues for elucidating the mechanism of EA function and provides a reference for finding new therapeutic targets for T2DM.

## Figures and Tables

**Table 1:** EA's intervention effect on FBG [($\bar{X}\pm \text{SD}$); *M* (*Q*1, *Q*2)].

Groups	(*n*)	FBG (mmol/L)		
		Before modelling	After modelling	14th day of EA treatment
Model group	10	8.96 ± 1.11	14.27 ± 2.17^*∗*^	22.88 (20.85, 24.91)^*∗*^^#^
Model + EA group	10	8.76 ± 1.17	14.77 ± 2.40^*∗*^	15.80 (14.09, 17.51)^Δ*∗*^
Normal group	10	8.51 ± 1.21	9.45 ± 1.10	9.54 (8.70, 10.37)^Δ^^#^

*Note.* ^*∗*^^,^^Δ^^,^^#^Statistical differences (*P* < 0.001) compared with the normal group, model group, and model + EA group, respectively.

**Table 2:** EA's intervention effect on the apoptosis rate of islet *β* cell [*M* (*Q*1, *Q*2)].

Groups	(*n*)	Apoptosis rate (%)
Model group	15	9.77 (3.48, 20.11)^*∗*^^#^
Model + EA group	15	5.31 (2.80, 8.63)^Δ^^∗^
Normal group	15	2.24 (0.00, 5.73)^Δ^^#^

*Note.* ^*∗*^^,^^Δ^^,^^#^Statistical differences (*P* < 0.05, *P* < 0.01, and *P* < 0.01, respectively) compared with the normal group, model group, and model + EA group, respectively.

**Table 3:** The top 10 upregulated and downregulated circRNAs ranked by fold changes.

circRNA	Circ ID	circRNA type	Fold change
*Upregulated* circRNAs			
chr11:103046379-103055289+	mmu_circ_0000325	Exonic	746.5258
chr6:47576536-47577667−	mmu_circ_0001471	Exonic	738.4114
chr10:40076564-40080807−	10_39800613_39796370_-4243|20.91|10|17	Exonic	649.1528
chr9:64160821-64161441−	9_64009248_64008628_-620|14.59|13|40	Exonic	503.0934
chr7:115659516-115662444−	7_122805958_122803030_-2928|21.29|10|16	Exonic	462.5214
chr11:72767580-72767898+	Novel	Sense overlapping	454.4070
chr4:84956276-84979521+	4_84625425_84602180_-23245|17.55|11|14	Exonic	413.8349
chr2:5052988-5054716−	2_4975762_4974034_-1728|15.56|5|6	Exonic	397.6061
chr12:91289489-91299077−	12_92537517_92527929_-9588|12.77|4|6	Exonic	381.3773
chr4:138146285-138151828+	Novel	Exonic	373.2629
*Downregulated* circRNAs			
chr6:119920110-119921028+	mmu_circ_0001516	Exonic	1492.2043
chr16:32950292-32961744+	mmu_circ_0000681	Exonic	637.69416
chr13:55213204-55238862+	13_55340223_55314565_-25658|19.14|4|4	exonic	573.92475
chr13:103884143-103897928−	13_104688008_104674223_-13785|17.98|20|70	Sense overlapping	561.17086
chr16:76330746-76352549−	mmu_circ_0000702	Exonic	548.41698
chr12:51619814-51661713−	mmu_circ_0000370	Exonic	535.66310
chr11:61736916-61745060−	Novel	Exonic	510.15533
chr17:24607437-24619570−	Novel	exonic	497.40145
chr1:165911288-165913349−	1_167843480_167841419_-2061|15.43|10|28	Exonic	484.64756
chr12:35078900-35086984+	12_35771656_35763572_-8084|19.56|22|64	Exonic	471.89368

**Table 4:** The top 10 GO annotations enrichment of upregulated differentially expressed host linear transcripts affected by EA intervention.

Gene function	Number of genes	*P*	False discovery rate	Differentially expressed genes
Cellular macromolecule metabolic process	73	<0.05	<0.05	CNPT1, ARID1A, STRN3, DKC1, GAB1, EZH2, MBD2, NCOA2, SOX6, ZEB1, etc.
Positive regulation of biological process	59	<0.05	<0.05	GAB1, EIF4G3, TGFBR2, PDE5A, ADCY9, ASPH, SULF1, CNOT1, CTCF, DNMT1, etc.
Macromolecule metabolic process	74	<0.05	<0.05	BRD4, CTCF, DNMT1, ETV6, EZH1, EZH2, HECTD1, UBE3A, KLHL7, KIF16B, etc.
Single-organism cellular process	93	<0.05	<0.05	CTCF, ZFP207, BRD4, ANP32B, GAB1, IARS, HECTD1, CNOT1, CLIC4, SULF1, etc.
Cellular metabolic process	78	<0.05	<0.05	MED13L, ACAACA, SDHC, ADCY9, ASCC3, STK39, RABGEF1, EIF4G3, ELF1, PLAGL1, etc.
Positive regulation of cellular process	53	<0.05	<0.05	GAB1, EIF4G3, TGFBR2, ADCY9, Adam10, ADNP, UBE3A, CTCELF1, SOX6, etc.
Single-organism process	98	<0.05	<0.05	OPTN, CTCF, ZFP207, BRD4, ANP32B, CIGALT1, UBE3A, TGFBR2, ST6GALNAC3, ST3GAL5, etc.
Regulation of cellular metabolic process	57	<0.05	<0.05	EIF4G3, RABGEF1, STK39, OPTN, KIF16B, RERE, ELF2, MLLT3, ATAD2, GPBP1, etc.
Regulation of macromolecule metabolic process	57	<0.05	<0.05	AGO2L, ELF1, ADNP, ARID4B, EIF4G3, TGFBR2, PLAGL1, CD46, PIK3CB, UBE2K, etc.
Regulation of metabolic process	60	<0.05	<0.05	BRD41, CTCF, DNMT1, ETV6, EZH1, EZH2, ATAD2B, PDGFD, ERBB2IP, DKC1, etc.

**Table 5:** The top 10 GO annotations enrichment of downregulated differentially expressed host linear transcripts affected by EA intervention.

Gene function	Number of genes	*P*	False discovery rate	Differentially expressed genes
Cellular metabolic process	90	<0.05	<0.05	POLA1, WHSC1, HIPK2, HOXB3, NFKB1, RAD23B, RAD52, FBXO18, ST3GAL6, PLCG2, etc.
Single-multicellular organism process	69	<0.05	<0.05	CBFB, SP3, PRKCA, MAP3K7, NRIP1, TNRC6C, IKZF1, NFKB1, NEK1, LATS2, etc.
Cellular macromolecule metabolic process	79	<0.05	<0.05	POLA1, WHSC1, HIPK2, HOXB3, NFKB1, PIAS1, ZBTB20, PRDM5, PHF14, EHMT1, etc.
Multicellular organism development	61	<0.05	<0.05	PRKCA, MAP3K7, NRIP1, CHM, ATF6, NEK1, LATS2, TSC2, IFT57, ARID11A, etc.
Metabolic process	95	<0.05	<0.05	CBFB, ARIH2, UBE2D2A, ANKIB1, TRIP12, ARID1A, STRN3, MAP3K7, PRKCA, DUSP3, etc.
Animal organ development	47	<0.05	<0.05	NRIP1, ATF6, SP3, IKZF1, NEK1, PLCG2, WHSC2, WHSC1, ZFPM1, MATR3, etc.
Primary metabolic process	88	<0.05	<0.05	PICALM, PRPSAP2, PIP5K1B, MECR, PLCG2, CD2AP, EP400, MLLT10, TTC7B, FAM126A, etc.
Regulation of cellular metabolic process	63	<0.05	<0.05	ZMYM5, EFEMP1, SP3, POU2F1, AFF3, IFT57, GON4L、TNRC6C, LARP4B, RPS6KB1, etc.
Regulation of macromolecule metabolic process	63	<0.05	<0.05	TCEA1, PLCG2, FBX018, RBM4, DUSP3, PRKCA, BOLL, TSC2, MATR3, PTPN22, etc.
Organic substance metabolic process	90	<0.05	<0.05	ARIH2, UBE2D2A, ANKIB1, TRIP12, STRN3, RSRC11, CDK13, SRBD1, ERI3, CCNE1, etc.

**Table 6:** The top 10 pathway enrichment of upregulated differentially expressed host linear transcripts affected by EA intervention.

Pathway	Selection counts	*P*	False discovery rate	Differentially expressed genes
Thyroid hormone signaling pathway	5	<0.05	<0.05	MED13, MED13L, NCOA2, PIK3CB, SLC16A10
Glycosphingolipid biosynthesis-ganglio series	2	<0.05	<0.05	ST3GAL5, ST6GALNAC3
cGMP-PKG signaling pathway	4	<0.05	<0.05	ADCY9, PDE5A, PIK3CB, ROCK1
Transcriptional misregulation in cancer	4	<0.05	<0.05	ETV6, MLLT3, TGFBR2, ZEB1
Choline metabolism in cancer	3	<0.05	<0.05	PCYT1A, PDGFD, PIK3CB
Rap1 signaling pathway	4	<0.05	<0.05	ADCY9, FYB, PDGFD, PIK3CB
Regulation of actin cytoskeleton	4	<0.05	<0.05	IQGAP2, PDGFD, PIK3CB, ROCK1
AMPK signaling pathway	3	<0.05	<0.05	ACACA, PIK3CB, RAB14
Platelet activation	3	<0.05	<0.05	ADCY9, PIK3CB, ROCK1
Regulation of lipolysis in adipocytes	2	<0.05	<0.05	ADCY9, PIK3CB

**Table 7:** The top 10 pathway enrichment of downregulated differentially expressed host linear transcripts affected by EA intervention.

Pathway	Selection counts	*P*	False discovery rate	Differentially expressed genes
Fc gamma R-mediated phagocytosis	5	<0.05	<0.05	PIP5K1B, PLCG2, PRKCA, RPS6KB1, CIN
HIF-1 signaling pathway	5	<0.05	<0.05	NFKB1, PDK1, PLCG2, PRKCA, RPS6KB1
Phosphatidylinositol signaling system	4	<0.05	<0.05	INPP4B, PIP5K1B, PLCG2, PRKCA
Choline metabolism in cancer	4	<0.05	<0.05	PIP5K1B, PRKCA, RPS6KB1, TSC2
Lysine degradation	3	<0.05	<0.05	EHMT1, NSD1, WHSC1
NOD-like receptor signaling pathway	3	<0.05	<0.05	ERBB2IP, MAP3K7, NFKB1
Thyroid hormone signaling pathway	4	<0.05	<0.05	MED12L, PLCG2, PRKCA, TSC2
mTOR signaling pathway	3	<0.05	<0.05	PRKCA, RPS6KB1, TSC2
Inositol phosphate metabolism	3	<0.05	<0.05	INPP4B, PIP5K1B, PLCG2
Proteoglycans in cancer	5	<0.05	<0.05	CTTN, KB1

**Table 8:** The predicted expression of top 10 upregulated circRNA-bound microRNAs affected by EA.

circRNA	miRNA	miRNA	miRNA	miRNA	miRNA
chr5:118593333-118593570+	mmu-miR-7028-5p	mmu-miR-762	mmu-miR-7079-5p	mmu-miR-6911-5p	mmu-miR-7662-5p
chr6:72128264-72132163+	mmu-miR-7669-3p	mmu-miR-5621-5p	mmu-miR-1943-5p	mmu-miR-7659-5p	mmu-miR-7662-3p
chr3:153411462-153411871−	mmu-miR-670-3p	mmu-miR-107-5p	mmu-miR-3089-3p	mmu-miR-103-1-5p	mmu-miR-103-2-5p
chr10:40076564-40080807−	mmu-miR-6954-5p	mmu-miR-3098-3p	mmu-miR-1966-5p	mmu-miR-708-5p	mmu-miR-7013-5p
chr11:86345860-86346028−	mmu-miR-335-3p	mmu-miR-7002-5p	mmu-miR-135b-5p	mmu-miR-6715-3p	mmu-miR-192-3p
chr9:99042524-99090320−	mmu-miR-7032-5p	mmu-miR-3069-5p	mmu-miR-103-1-5p	mmu-miR-103-2-5p	mmu-miR-1934-3p
chr16:4285593-4286065−	mmu-miR-204-3p	mmu-miR-191-3p	mmu-miR-7a-5p	mmu-miR-1953	mmu-miR-7054-5p
chr1:13221307-13286025−	mmu-miR-762	mmu-miR-7007-5p	mmu-miR-337-3p	mmu-miR-421-5p	mmu-miR-7648-3p
chr18:10119885-10132272−	mmu-miR-7660-3p	mmu-miR-6992-3p	mmu-miR-1903	mmu-miR-6380	mmu-miR-7665-5p
chr3:122747889-122748477+	mmu-miR-6339	mmu-miR-1954	mmu-miR-6912-5p	mmu-miR-500-5p	mmu-miR-7033-5p

**Table 9:** The predicted expression of top 10 downregulated circRNA-bound microRNAs affected by EA.

circRNA	miRNA	miRNA	miRNA	miRNA	miRNA
chr2:24847944-24863908−	mmu-miR-206-5p	mmu-miR-7038-3p	mmu-miR-145a-3p	mmu-miR-7084-3p	mmu-miR-8112
chr19:24346237-24360151−	mmu-miR-1903	mmu-miR-6982-5p	mmu-miR-6387	mmu-miR-107-5p	mmu-miR-3068-5p
chr17:24607437-24619570−	mmu-miR-6998-5p	mmu-miR-1906	mmu-miR-7001-5p	mmu-miR-214-3p	mmu-miR-7222-3p
chr11:86532772-86535460−	mmu-miR-29a-5p	mmu-miR-5623-3p	mmu-miR-6395	mmu-miR-328-3p	mmu-miR-16-1-3p
chr2:71875406-71880110+	mmu-miR-29a-5p	mmu-miR-6937-5p	mmu-miR-192-3p	mmu-miR-7075-5p	mmu-miR-494-3p
chr12:40124567-40128035−	mmu-miR-127-5p	mmu-miR-3095-5p	mmu-miR-6973b-3p	mmu-miR-6365	mmu-miR-7236-3p
chr8:82068932-82071835+	mmu-miR-7092-3p	mmu-miR-7090-3p	mmu-miR-761	mmu-miR-6934-5p	mmu-miR-3966
chr8:117555975-117558116+	mmu-miR-6918-5p	mmu-miR-7014-5p	mmu-miR-3079-5p	mmu-miR-212-5p	mmu-miR-432
chr11:108012627-108014381−	mmu-miR-1903	mmu-miR-7065-3p	mmu-miR-7215-3p	mmu-miR-7009-5p	mmu-miR-3074-2-3p
chr3:135655500-135669339−	mmu-miR-145a-5p	mmu-miR-34a-5p	mmu-miR-216a-3p	mmu-miR-6964-3p	mmu-miR-3066-5p

## Data Availability

The data used to support the findings of this study are included within the article and the supplementary information files. The sequencing data have been uploaded to the Gene Expression Omnibus (GEO) database (GEO ID: GSE133665).

## Supplementary Materials

Supplementary Materials Table S1: the primers used in the present study. Table S2: circRNA expression profiling identified in the plasma exosomes of three groups. Table S3: upregulated and downregulated differentially expressed circRNAs among three groups. Table S4: pathways related with upregulated and downregulated host linear transcripts among three groups. Table S5: each circRNA and its potential complementary binding miRNAs. Figure S1: plasma exosomes confirmed by western blotting (A), transmission electron microscopy (B), and NTA (C). Figure S1A shows that exosome-enriched markers CD9, TSG101, and CD63 were abundant in plasma exosomes and that no calnexin was observed. Transmission electron microscopy confirmed the shape and morphology of the isolated exosomes. Through NTA, we obtained the total exosome particle number (particles per ml). The average exosome number was 8.8 × 108 ml. The vesicle size in the sample was mainly distributed in the range of 70–200 nm, which accorded with the size range of exosomes. The particle size range of 91.6% was about 152.8 nm. Figure S2 shows the gene ontology (GO) annotation of downregulated host linear transcripts among three groups. Figure S3 shows the gene ontology (GO) annotation of upregulated host linear transcripts among three groups.
