# Supplementary figures and images for: Determination of Electroacupuncture Effects on circRNAs in Plasma Exosomes in Diabetic Mice: An RNA-Sequencing Approach
Source: Evid Based Complement Alternat Med. 2019 Sep 24;2019:7543049. doi: 10.1155/2019/7543049 (PMC6778869; doi:10.1155/2019/7543049)

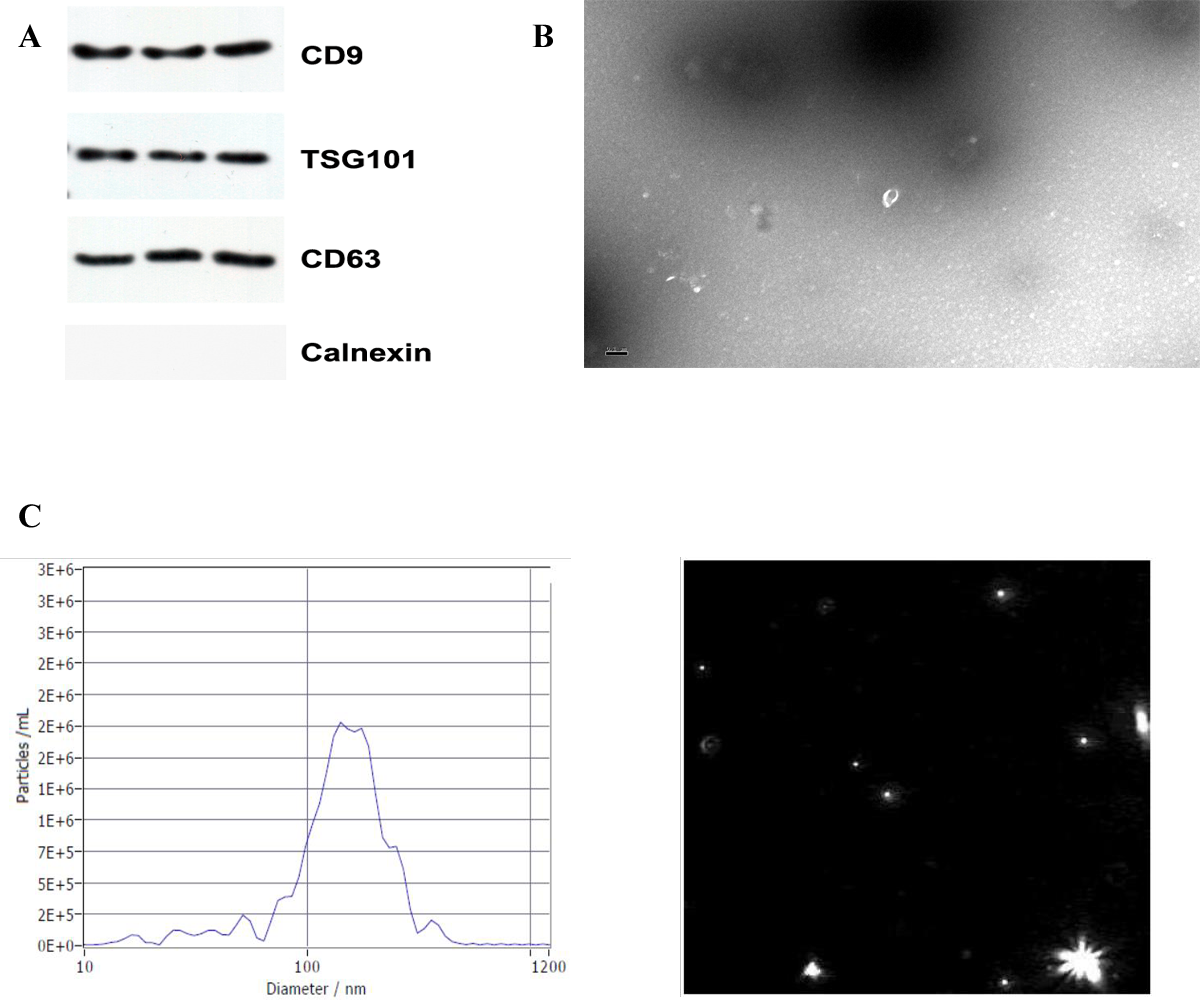

Supplement: Supplementary Materials — Table S1: the primers used in the present study. Table S2: circRNA expression profiling identified in the plasma exosomes of three groups. Table S3: upregulated and downregulated differentially expressed circRNAs among three groups. Table S4: pathways related with upregulated and downregulated host linear transcripts among three groups. Table S5: each circRNA and its potential complementary binding miRNAs. Figure S1: plasma exosomes confirmed by western blotting (A), transmission electron microscopy (B), and NTA (C). Figure S1A shows that exosome-enriched markers CD9, TSG101, and CD63 were abundant in plasma exosomes and that no calnexin was observed. Transmission electron microscopy confirmed the shape and morphology of the isolated exosomes. Through NTA, we obtained the total exosome particle number (particles per ml). The average exosome number was 8.8 × 108 ml. The vesicle size in the sample was mainly distributed in the range of 70–200 nm, which accorded with the size range of exosomes. The particle size range of 91.6% was about 152.8 nm. Figure S2 shows the gene ontology (GO) annotation of downregulated host linear transcripts among three groups. Figure S3 shows the gene ontology (GO) annotation of upregulated host linear transcripts among three groups. [file 7543049.f1.zip › 7543049.f1/Supplementary Figure S1.tif]

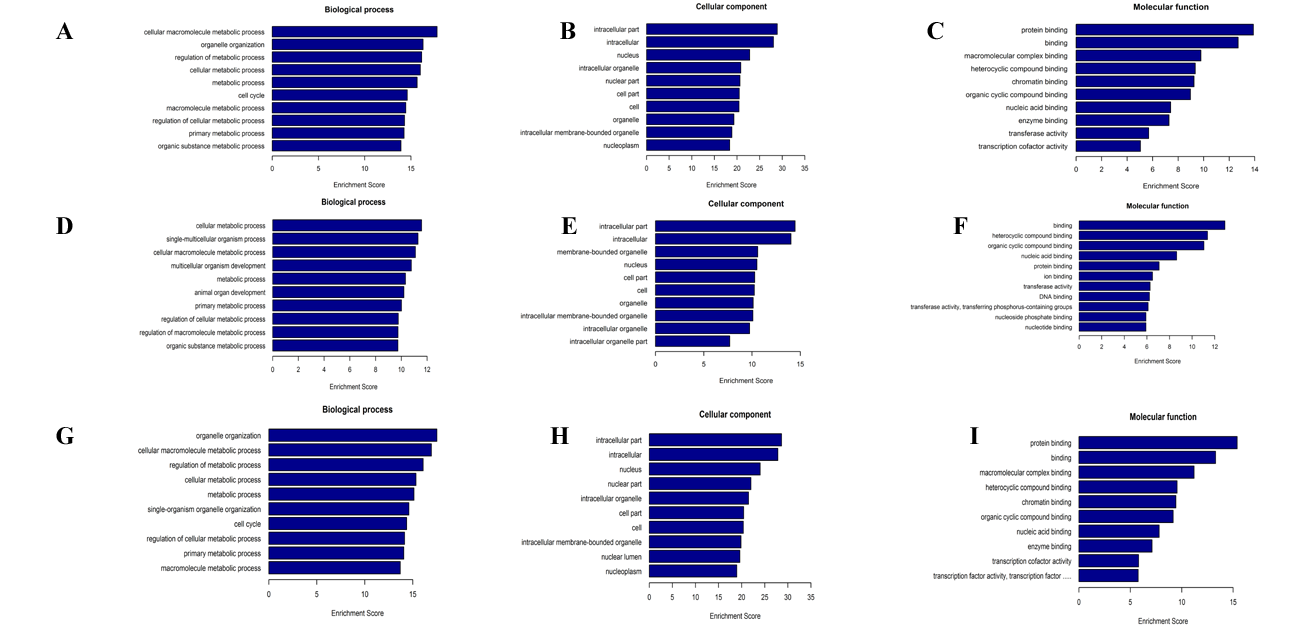

Supplement: Supplementary Materials — Table S1: the primers used in the present study. Table S2: circRNA expression profiling identified in the plasma exosomes of three groups. Table S3: upregulated and downregulated differentially expressed circRNAs among three groups. Table S4: pathways related with upregulated and downregulated host linear transcripts among three groups. Table S5: each circRNA and its potential complementary binding miRNAs. Figure S1: plasma exosomes confirmed by western blotting (A), transmission electron microscopy (B), and NTA (C). Figure S1A shows that exosome-enriched markers CD9, TSG101, and CD63 were abundant in plasma exosomes and that no calnexin was observed. Transmission electron microscopy confirmed the shape and morphology of the isolated exosomes. Through NTA, we obtained the total exosome particle number (particles per ml). The average exosome number was 8.8 × 108 ml. The vesicle size in the sample was mainly distributed in the range of 70–200 nm, which accorded with the size range of exosomes. The particle size range of 91.6% was about 152.8 nm. Figure S2 shows the gene ontology (GO) annotation of downregulated host linear transcripts among three groups. Figure S3 shows the gene ontology (GO) annotation of upregulated host linear transcripts among three groups. [file 7543049.f1.zip › 7543049.f1/Supplementary Figure S2.tif]

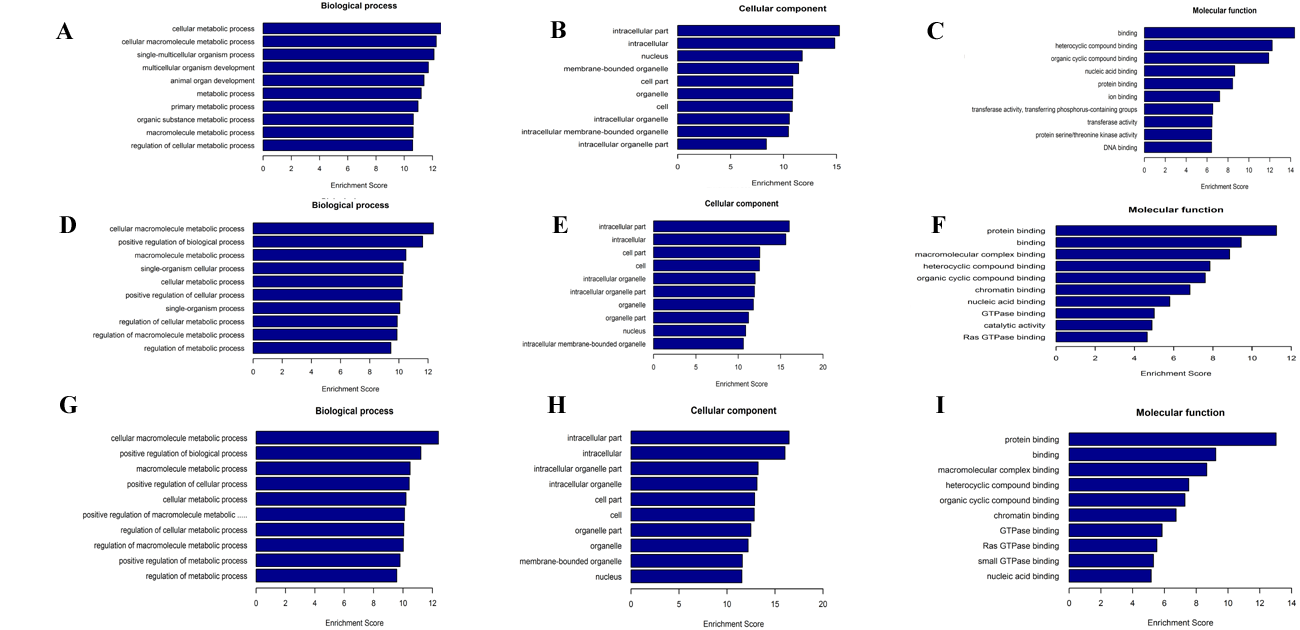

Supplement: Supplementary Materials — Table S1: the primers used in the present study. Table S2: circRNA expression profiling identified in the plasma exosomes of three groups. Table S3: upregulated and downregulated differentially expressed circRNAs among three groups. Table S4: pathways related with upregulated and downregulated host linear transcripts among three groups. Table S5: each circRNA and its potential complementary binding miRNAs. Figure S1: plasma exosomes confirmed by western blotting (A), transmission electron microscopy (B), and NTA (C). Figure S1A shows that exosome-enriched markers CD9, TSG101, and CD63 were abundant in plasma exosomes and that no calnexin was observed. Transmission electron microscopy confirmed the shape and morphology of the isolated exosomes. Through NTA, we obtained the total exosome particle number (particles per ml). The average exosome number was 8.8 × 108 ml. The vesicle size in the sample was mainly distributed in the range of 70–200 nm, which accorded with the size range of exosomes. The particle size range of 91.6% was about 152.8 nm. Figure S2 shows the gene ontology (GO) annotation of downregulated host linear transcripts among three groups. Figure S3 shows the gene ontology (GO) annotation of upregulated host linear transcripts among three groups. [file 7543049.f1.zip › 7543049.f1/Supplementary Figure S3.tif]
